# Dynamic behavior of suture-anastomosed arteries and implications to vascular surgery operations

**DOI:** 10.1186/1475-925X-14-1

**Published:** 2015-01-06

**Authors:** Panayiotis C Roussis, Antonios E Giannakopoulos, Haralambia P Charalambous, Demetra C Demetriou, Georgios P Georghiou

**Affiliations:** Department of Civil & Environmental Engineering, University of Cyprus, Nicosia, CY-1678 Cyprus; Department of Civil Engineering, University of Thessaly, Volos, GR-38334 Greece; Cardiothoracic Surgery Department, American Medical Center, Nicosia, CY-1311 Cyprus; Sackler Faculty of Medicine, Tel Aviv University, Tel Aviv, 69978 Israel

**Keywords:** End-to-end anastomosis, Anastomotic gap, Sutures, Arterial-wall stress, Suture stress, Failure criteria

## Abstract

**Background:**

Routine vascular surgery operations involve stitching of disconnected human arteries with themselves or with artificial grafts (arterial anastomosis). This study aims to extend current knowledge and provide better-substantiated understanding of the mechanics of end-to-end anastomosis through the development of an analytical model governing the dynamic behavior of the anastomotic region of two initially separated arteries.

**Methods:**

The formulation accounts for the arterial axial-circumferential deformation coupling and suture-artery interaction. The proposed model captures the effects of the most important parameters, including the geometric and mechanical properties of artery and sutures, number of sutures, loading characteristics, longitudinal residual stresses, and suture pre-tensioning.

**Results:**

Closed-form expressions are derived for the system response in terms of arterial radial displacement, anastomotic gap, suture tensile force, and embedding stress due to suture-artery contact interaction. Explicit objective functionalities are established to prevent failure at the anastomotic interface.

**Conclusions:**

The mathematical formulation reveals useful interrelations among the problem parameters, thus making the proposed model a valuable tool for the optimal selection of materials and improved functionality of the sutures. By virtue of their generality and directness of application, the findings of this study can ultimately form the basis for the development of vascular anastomosis guidelines pertaining to the prevention of post-surgery implications.

## Background

Predictive medicine and therapeutic decision-making requires thorough understanding of the human biological activities in order to develop a proper physical model and obtain the optimal solution of the problem. Vascular surgery operations treat vascular diseases, traffic-related and other serious injuries entailing violent artery damage. Among the vascular disorders, atherosclerosis and aneurysms are the most frequently encountered. During a typical arterial reconstruction, the diseased artery segment is removed, and the healthy segments are stitched together, either directly or through the insertion of an artificial graft (end-to-end anastomosis). In any case, the mechanical behavior of the anastomotic region is in principle comparable, on account of the fact that modern grafts tend to exhibit similar geometric and stiffness characteristics with those of arteries.

Several studies have examined the induced arterial-wall stresses in vascular anastomosis models [1–7]. However, most of them limit their research to specific arterial geometries, while others ignore the stress concentrations due to suture-artery interaction, or the important axial-circumferential deformation coupling in the artery response. Moreover, most of the published studies rely solely on finite element analyses rather than on analytical models of end-to-end and end-to-side anastomosis.

In particular, Ballyk et al. [1] studied an end-to-end and an end-to-side anastomosis by use of finite-element analysis, with the sutures modeled as discrete points along the suture line. The expected stress concentration of the stitching area resulted in excessive values due to the point-like modelling approach as such. Leuprecht et al. [2] and Perktold et al. [3] utilized three end-to-side anastomosis models, each one concerning a different technique. Their finite-element analysis yielded the wall shear stresses and maximum principal stresses for each case. In particular, the latter study modeled the stitches in detail using three-dimensional elements for the junction. In another finite element study, Cacho et al. [6] investigated the effect of the insertion angle and incision length of coronary arterial bypass models, though without modelling explicitly the response of individual stitches. More recently, Schiller et al. [7] studied an end-to-end anastomosis by using a fluid–structure coupling algorithm, with the sutures simulated as an anastomotic interface.

In addition to theoretical studies, which on one hand are limited and on the other hand require numerical methods to calculate the response [8], several experimental studies have been performed over the years (see for example [9–13]) to investigate the compliance of the anastomotic region. Lyman et al. [9] found that vascular grafts should have compliance approximately equal to that of the host artery, and that compliance of synthetic grafts may decrease with time. Hasson et al. [10] investigated an end-to-end anastomosis between isocompliant arterial grafts from dogs and found that a para-anastomotic hypercompliant zone (PHZ), which promotes subintimal hyperplasia (SIH), exists near the suturing region. The PHZ is also a zone of increased cyclic stretch. The compliance at this region increases up to 50% compared to the compliance away from the stitching region. In a later study, Hasson et al. [11] concluded that the suture technique affects significantly the compliance of the anastomotic region. In particular, they showed that the PHZ phenomenon occurs more frequently for anastomosis of the continuous stitching technique than the interrupted stitching technique. In addition, they observed that increased longitudinal stress of the arterial vessel reduce the compliance. This phenomenon can be justified from the fact that longitudinal pre-stress affects the mechanical properties of dog arteries [14]. Ulrich et al. [13] investigated an end-to-end anastomosis between pig aortic grafts and found that PHZ does not exist for this case. They also suggested that the main factor affecting the anastomotic response is the suture line itself.

Evidently, little work has been published on the dynamic analysis of the stitched anastomotic region. Indeed, related review articles clearly point out the lack of such analyses [15]. In addressing this need, this paper proposes a comprehensive analytical model based on structural analysis, aiming to provide a better understanding of the dynamic response of end-to-end arterial anastomosis, with emphasis on the suture-artery interaction and the axial-circumferential deformation coupling in the artery response. This study seeks to extend current knowledge and provide practical suggestions for the optimal selection of materials and improved functionality of the sutures in vascular surgery operations.

## Methods

This section presents the mathematical formulation governing the dynamic behavior of end-to-end arterial anastomosis. The system response is described in terms of the radial displacement of artery, the anastomotic gap, the suture tensile force, and the embedding stress due to suture-artery contact interaction. Based on these response quantities, comprehensive objective functionalities are established in order to prevent failure at the anastomotic interface.

### Arterial model

From the mechanics point of view, the human arterial system can be idealized as a system of cylindrical elastic pipes that transport blood under pressure provided from the heart (dynamic loading). The arterial tissue is heterogeneous, consisting of three inhomogeneous layers. Its mechanical properties depend on the artery location, age, disease, and other physiological states [16, 17]. In general, the mechanical behavior of the arterial tissue does not obey Hooke’s law [18, 19], exhibiting anisotropic nonlinear behavior for finite deformations. Moreover, the response of biological tissues is affected by the existence of residual stresses [20]. In this study, the arterial wall is assumed homogeneous and its mechanical response linear elastic. The assumed elastic properties in the model incorporate in an average sense the tangential stiffness, the anisotropy, the inhomogeneity, and the residual stresses of the artery walls.

**Figure 1:**
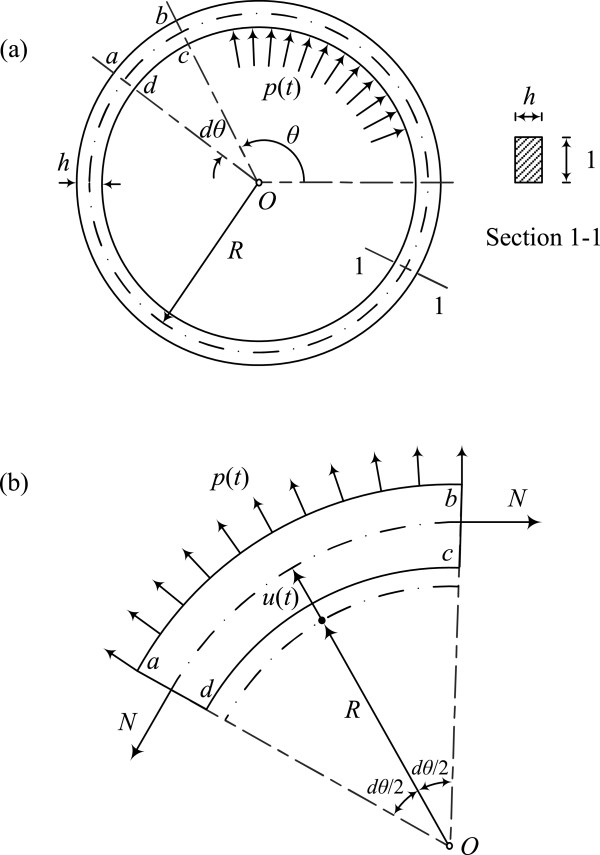
**Idealized arterial system. (a)** Arterial model, **(b)** typical element of circular ring.

In this study, the blood vessel is modeled as an elastic cylindrical pipe with wall thickness *h* and radius *R* (Figure 1(a)). The mathematical formulation developed herein is based on the following assumptions: (a) the arterial wall thickness is small compared to the radius of the centerline of the ring therefore the radial stresses are not considered; (b) the centerline of the ring in the undeformed state forms a full circle with radius *R*_;_ (c) the cross-section is axially symmetric and constant around the circle, implying that the arterial wall has constant thickness; (d) no boundary constraints are applied on the ring; (e) the effects of rotary inertia and shear deformation are neglected; (f) the arterial tissue consists of a single homogeneous layer and behaves as a simple orthotropic linear elastic material (the mechanical properties in the radial and circumferential directions − which are the same − differ from those in the longitudinal direction, ignoring the Poisson effect in the orthotropy constitutive law); and (g) viscous effects are ignored. The simplified orthotropic model adopted in this work utilizes two elastic constants *E*_*θ*_ and *E*_*L*_ which are the plane strain elastic moduli suggested by Schajer et al. [21], in the circumferential and the longitudinal directions respectively.

#### Response to general dynamic loading

Under the assumption of axial symmetry, the system undergoes in-plane extensional vibration due to a uniformly-distributed wall pressure *p*(*t*). The differential equation governing the radial displacement *u*(*t*) of the vibrating arterial ring can be derived by considering the equilibrium of forces acting on an element of the circular ring (Figure 1(b)). The resulting equation is (see Appendix A1)
1

where *ρ* denotes the density of the arterial tissue, *h* the wall thickness, *R* the radius of the undeformed centerline of the ring, and *E*_*θ*_ the arterial Young’s modulus in the circumferential direction.

The first term in equation (1) represents the radial inertia force acting on element *abcd* of the circular ring, while the second term represents the circumferential tensile force developed on the element cross section assuming linear-elastic behavior.

Equation (1) is identical to the classical second-order differential equation governing the response of an undamped single-degree-of-freedom system to arbitrary force. The circular frequency of the system is readily obtained as
2

#### Response to pulse-type loading

**Figure 2:**
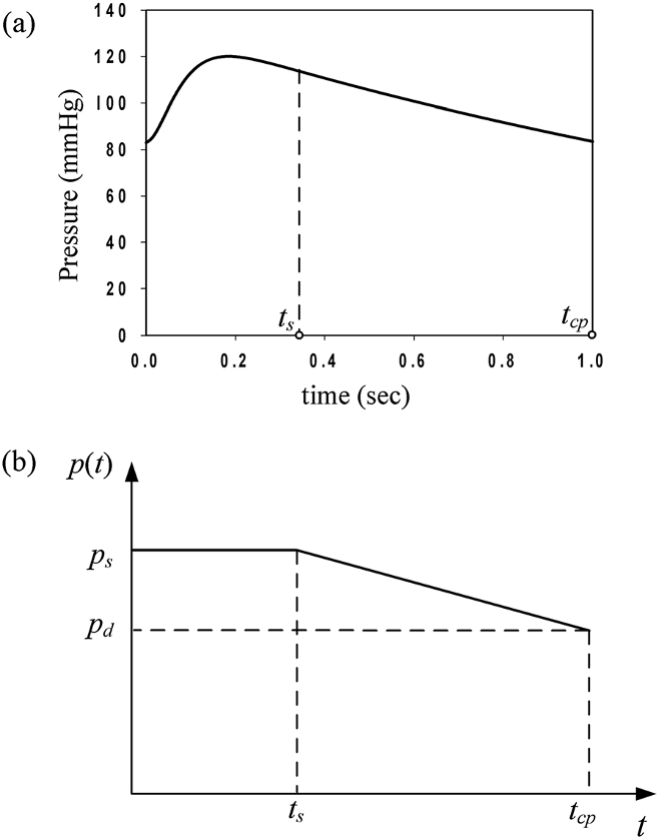
**Blood pressure time-profiles. (a)** Typical aortic pressure time-profile following Zhong et al. [22], **(b)** arterial pulse time-history approximation. (100 mmHg = 13.33 kPa).

A normal cardiac cycle consists of two major functional periods: systolic and diastolic. Figure 2(a) shows the aortic pressure–time profile as proposed by Zhong et al. [22]. The time interval 0 ≤ *t* ≤ *t*_*s*_ represents the aortic systolic phase, during which the arterial walls inflate due to the maximum overstress pressure. The time interval *t*_*s*_ < *t* ≤ *t*_*cp*_ represents the aortic diastolic phase.

During a vascular surgery operation the blood flow is interrupted. The first loading cycle, immediately after the flow is restored, is approximated in this study by the loading shown in Figure 2(b). In this case, the internal pressure is abruptly increased from zero to the maximum systolic pressure. The assumed loading is expressed mathematically as

3

where *p*_*s*_ is the maximum systolic pressure, *p*_*d*_ the diastolic pressure, *t*_*s*_ the systolic-phase duration, and *t*_*cp*_ the total duration of the cardiac pulse.

The radius *R* is measured at zero blood pressure and in vivo length, implying the artery is in its pre-stressed state. During surgery, longitudinal residual stresses are released, forcing the artery to decrease its length and increase its diameter. When subsequently the stitching takes place, the arterial diameter and length return to their prior condition. The residual-stress effect is taken into account as an initial displacement *u*(0) = *u*_0_ (and so the far-field stresses can be inserted), equal to the difference of the increased radius (relieved from axial residual stresses) and radius *R*, and initial velocity .

The total response of the system as a function of time is obtained as (see Appendix A2)
4

in which the first term (free-vibration response) is associated with the residual-stress effect and the second term is associated with the response to the assumed pulse-type loading.

The static response of the system, which corresponds to the displacement caused when the maximum pressure *p*_*s*_ is applied statically, is identified as
5

Of particular interest is the maximum arterial displacement, which is associated with the critical response of the anastomotic region. The maximum displacement occurs either in the time interval 0 ≤ *t* ≤ *t*_*s*_ or *t*_*s*_ < *t* ≤ *t*_*cp*_, depending on the system natural period *T*_*n*_ = 2*π*/*ω*_*n*_ and the loading characteristics. The overall maximum displacement can be calculated from the following expression (for |*u*_0_|/*u*_*st*_ < 1): (see Appendix A3)
6

where *t*_1_ is the time instant corresponding to the maximum response in the diastolic phase, given by
7

in which
89

**Figure 3:**
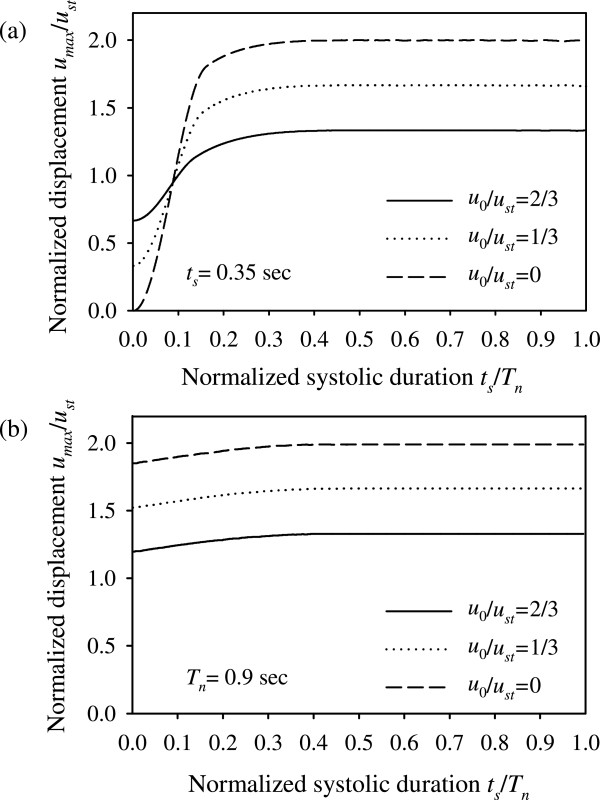
**Normalized maximum displacement as a function of** ***t***_***s***_ ***/T***_***n***_ **and the parameter** ***u***_***0***_ ***/u***_***st***_ **.** Plots for **(a)** *t*_*s*_ = 0.35 sec, **(b)** *T*_*n*_ = 0.9 sec.

Figure 3 plots the normalized maximum deformation *u*_max_/*u*_*st*_ as a function of the ratio *t*_*s*_/*T*_*n*_ for different values of initial displacement, and for typical values of diastolic pressure, maximum systolic pressure, and cardiac pulse duration (*p*_*d*_ = 80 mmHg, *p*_*s*_ = 120 mmHg, *t*_*cp*_ = 1 sec). In particular, Figure 3(a) plots the normalized response for a typical cardiac pulse with fixed systolic-phase duration, *t*_*s*_ = 0.35 sec, and varying system natural period *T*_*n*_. As can be seen in Figure 3(a), the response exhibits an ascending curved profile for low values of *t*_*s*_/*T*_*n*_, reaching practically a plateau for high values of *t*_*s*_/*T*_*n*_. .he threshold value of *t*_*s*_/*T*_*n*_ that defines the boundary between the ascending part and the plateau depends on the loading characteristics. For the parameters used in Figure 3(a), the threshold value of *t*_*s*_/*T*_*n*_ is approximately 0.4. Figure 3(b) plots the normalized response for fixed natural period *T*_*n*_ = 0.9 sec and varying systolic-phase duration *t*_*s*_. The high value of natural period (*T*_*n*_ = 0.9 sec) implies severely damaged artery walls, with the artery elasticity modulus practically tending to zero.

### Anastomosis model

**Figure 4:**
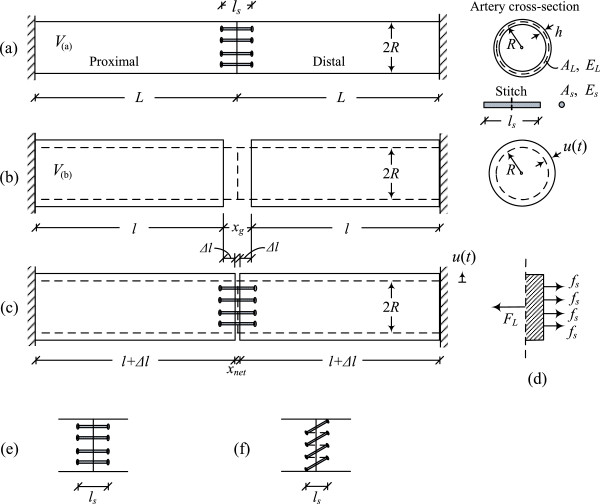
**End-to-end anastomosis analysis between isocompliant blood vessels. (a)** Anastomosis model (at-rest state); the artery is clamped at the far ends and no pressure is transmitted at this stage since the artery is emptied from the blood, **(b)** unrestrained deformed state of artery (without sutures); the blood volume is conserved, **(c)** deformed state of anastomotic region due to dynamic loading, **(d)** forces acting on end-element of artery segment, **(e)** interrupted stitching scheme, **(f)** continuous stitching scheme.

A schematic of the end-to-end anastomosis model considered in this study is shown in Figure 4(a). The proximal and distal artery segments are connected together with a total of *N*_*s*_ stitches. Each artery segment has length *L*, radius *R*, and Young’s modulus in the longitudinal direction *E*_*L*_. The stitches have radius *r*_*s*_, cross-sectional area *A*_*s*_ = *πr*_*s*_^2^, and Young’s modulus *E*_*s*_. The suture material is legitimately considered to be linear elastic for elongations up to 20% [23]. The distance between stitching holes that are symmetrically located across the separation plane is denoted by *l*_*s*_ (with the assumption that 2*L* ≫ *l*_*s*_). Different stitching patterns are considered, resulting in different suture loading. Figure 4(e) depicts the *interrupted* stitching scheme, whereas Figure 4(f) depicts the *continuous* (*running*) stitching scheme. The particular loading condition associated with each stitching scheme is accounted for in the analysis by means of a participation factor *a*. The interrupted stitching scheme corresponds to a maximum participation factor *a* = 2, whereas the continuous stitching scheme (with diagonal at 45° angle), corresponds to participation factor *a* = 1.707. The participation factor is derived from the local equilibrium of forces at the suture line that passes without friction through the stitch hole. The participation factor indicates the alignment of the stitches along the longitudinal direction (the remaining part (2 − *a*) indicates that the system is in torsion with limited relevance to the present problem). Moreover, the stitching holes and the suture are considered to have almost equal diameters. Therefore, the suture segment penetrating the arterial wall is almost undeformable, due to friction forces developed between the arterial wall and the suture. The model also considers the pre-tensioning of stitches [24], denoted herein by the force . This is the force exerted by the surgeon in tying the knot of the suture.

#### Objective functionalities

The interaction of sutures with the arterial tissue may lead to post-surgery complications. The undesirable conditions can be described in three failure scenarios: suture failure, arterial-wall tearing, and thrombosis (due to blood leaking) at the anastomotic interface. Suture failure is caused when the maximum tensile force of the suture, *f*_*s*_, exceeds the suture strength or leads to slip or relaxation of the knots that bind the stitches together [25]. Note that it is possible that suture failure may occur due to suture gradual deterioration with time [26]. Arterial-wall rupture or injury may be caused when the embedding stresses, *σ*_*s*_, due to suture-artery contact interaction (at the stitching holes) exceed the limit value of artery-wall shear strength. Thrombosis may be caused if the distance between the edges of the two anastomosed artery segments, *x*_*net*_, exceeds the typical size of a few red blood cells, leading to internal bleeding.

In order to avoid failure altogether, the following objective functionalities must be satisfied:
101112

In addition to the above objective functionalities, the following geometric constraint must be satisfied to assure adequate stitching spacing:
13

It should be noted that mechanical changes to the arterial walls and sutures may occur over a time span of several weeks after surgery. In particular, the wall thickness of the sutured artery may decrease with time, as is the case of the inflammatory response after surgery. Moreover, the elastic properties and strength of the artery may change with time due to chemical change of the suture and its interaction with the arteries [26]. Such long-term implications lead to lower values of the elastic and strength properties of the arterial walls and suture materials.

#### Suture-artery interaction

On account of the fact that blood is an incompressible fluid, the radial and longitudinal modes of arterial response are coupled. Under the applied blood pressure, the artery distends radially by *u*(*t*), and, in order for the blood volume to be maintained, its axial length is decreased from *L* to *l*, resulting in the formation of a gap *x*_*g*_ (Figure 4(b)). Conservation of the blood volume means that the cylindrical volume *V*_(*a*)_ = *V*_(*b*)_ (Figures 4(a,b)). Then, the decreased anastomosis length at any time *t* is given by
14

The separation distance *l*(*t*) given by equation (14) implies the following solid–fluid interaction procedure: (a) the blood volume fills the two parts of the anastomosis after completing the stitching, and (b) pressure is applied leading to contraction along the length of the initially emptied artery.

The gap developed in the unrestrained (without sutures) state of the artery (Figure 4(b)), is then determined as the difference between the initial length of the artery (2*L*) and the length of the unrestrained deformed state (2*l*):

15Therefore, the resulting net gap developed in the restrained (with sutures) anastomotic region (Figure 4(c)) can be derived from
16

where Δ*l* is the tensile deformation due to the stitching stiffness.

The tensile forces developed in the suture and arterial tissue (Figure 4(d)) are given respectively by
1718

where *ϵ*_*s*_ is the suture strain, *ϵ*_*L*_ is the strain of one artery segment, and *A*_*L*_ is the cross-sectional area of the artery.

The tensile deformation Δ*l* can be derived from equilibrium of forces in the axial direction, *F*_*L*_(*t*) = *aN*_*s*_*f*_*s*_(*t*), yielding (see Appendix A4)
19

Substituting equations (15) and (19) into equation (16), we obtain the net gap between the anastomosed artery segments as
20

Note that a gap across the anastomotic interface will be formed only if the tension developed in the arterial tissue exceeds the total suture pre-tension.

Upon calculating the anastomotic gap *x*_*net*_, the suture tensile force *f*_*s*_ developed in each stitch can be readily obtained from equation (17). In addition, embedding stresses *σ*_*s*_ are developed because of suture-artery contact interaction at the stitching holes. The embedding stress induced on the arterial wall is approximated [27] by
21

It is worth noting that, although based on a linear-elastic model, the system response depends on a considerable number of parameters. In particular, the solution contains as many as seventeen input parameters (*L*, *R*, *N*_*s*_, *h*, *E*_*θ*_, *E*_*L*_, *p*_*s*_, *p*_*d*_, *t*_*s*_, *t*_*cp*_, *ρ*, *u*_0_, *l*_*s*_, *E*_*s*_, *r*_*s*_, *a*, ) related to the geometric and mechanical properties of sutures and arterial walls, the number of sutures, the loading characteristics, the longitudinal residual stresses, and suture pre-tensioning.

For completeness, the general solution of an artery/graft end-to-end anastomosis is presented in Appendix B.

## Results and discussion

The three response quantities of interest, the anastomotic gap *x*_*net*_, the suture tensile force *f*_*s*_, and the embedding stress *σ*_*s*_, are directly connected to the aforementioned failure modes. On normalizing by 2 *L*, *E*_*L*_*h*^2^ and *E*_*L*_ respectively, equations (20), (17) and (21) become
222324

**Figure 5:**
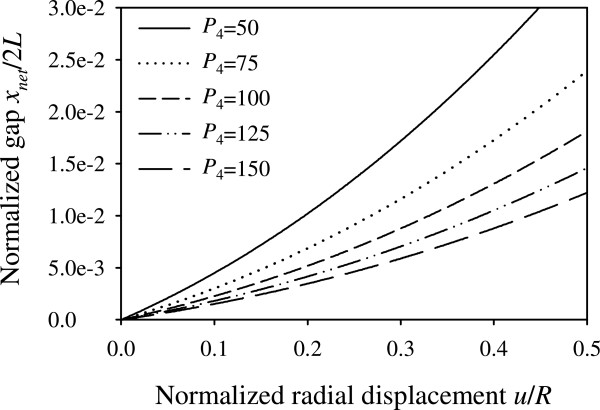
**Normalized anastomotic gap versus normalized radial displacement.** Plots for different values of product  and for .

**Figure 6:**
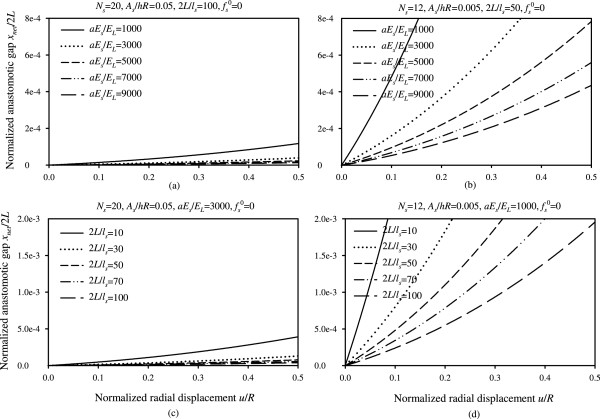
**Normalized anastomotic gap versus normalized radial displacement. (a)** Plots for different values of parameter *aE*_*s*_/*E*_*L*_ and for *N*_*s*_ = 20, *A*_*s*_/*hR* = 0.05, 2*L*/*l*_*s*_ = 100, , **(b)** Plots for different values of parameter *aE*_*s*_/*E*_*L*_ and for *N*_*s*_ = 12, *A*_*s*_/*hR* = 0.005, 2*L*/*l*_*s*_ = 50, , **(c)** Plots for different values of parameter 2*L*/*l*_*s*_ and for *N*_*s*_ = 20, *A*_*s*_/*hR* = 0.05, *aE*_*s*_/*E*_*L*_ = 3000, , **(d)** Plots for different values of parameter 2*L*/*l*_*s*_ and for *N*_*s*_ = 12, *A*_*s*_/*hR* = 0.005, *aE*_*s*_/*E*_*L*_ = 1000, .

**Figure 7:**
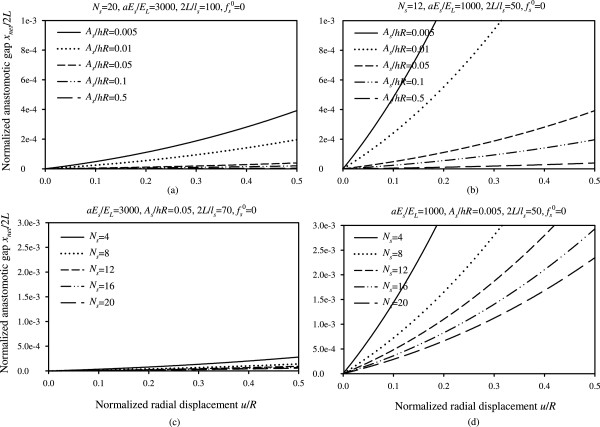
**Normalized anastomotic gap versus normalized radial displacement. (a)** Plots for different values of parameter *A*_*s*_/*hR* and for *N*_*s*_ = 20, *aE*_*s*_/*E*_*L*_ = 3000, 2*L*/*l*_*s*_ = 100, , **(b)** Plots for different values of parameter *A*_*s*_/*hR* and for *N*_*s*_ = 12, *aE*_*s*_/*E*_*L*_ = 1000, 2*L*/*l*_*s*_ = 50, , **(c)** Plots for different values of parameter *N*_*s*_ and for *aE*_*s*_/*E*_*L*_ = 3000, *A*_*s*_/*hR* = 0.05, 2*L*/*l*_*s*_ = 70, , **(d)** Plots for different values of parameter *N*_*s*_ and for *aE*_*s*_/*E*_*L*_ = 1000, *A*_*s*_/*hR* = 0.005, 2*L*/*l*_*s*_ = 50, .

**Figure 8:**
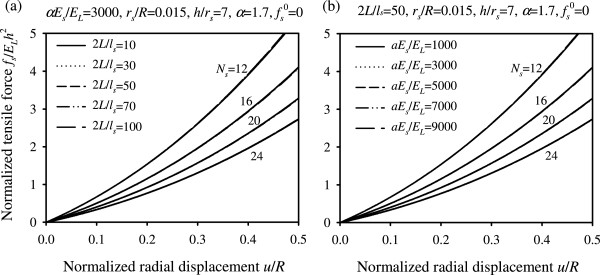
**Normalized tensile force in each stitch versus normalized radial displacement. (a)** Plots for different values of parameters 2*L*/*l*_*s*_ and *N*_*s*_ **(b)** Plots for different values of parameters *a* *E*_s_/*E*_*L*_ and *N*_*s*_. ().

**Figure 9:**
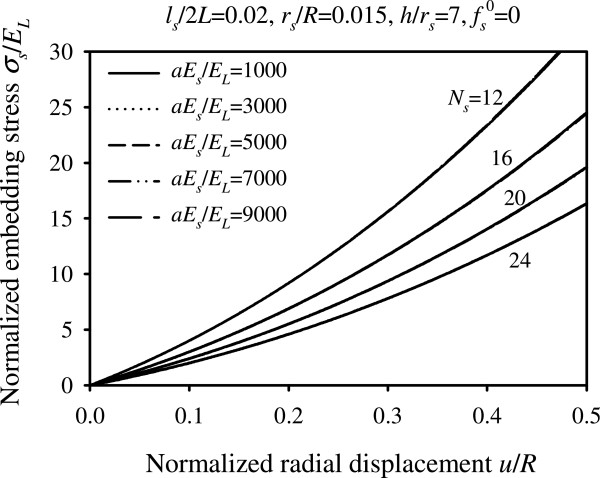
**Normalized embedding stress versus normalized radial displacement.** Plots for different values of parameter *aE*_*s*_/*E*_*L*_, *N*_*s*_ and for .

From equations (22), (23), (24), we observe that the seventeen input parameters of the problem can be reduced into five dimensionless parameters, namely *aE*_*s*_/*E*_*L*_, *L*/*l*_*s*_, *N*_*s*_*r*_*s*_/*R*, *r*_*s*_/*h*, . The normalized response quantities for parameter values varied within the physiological range are presented graphically in Figures 5, 6, 7, 8 and 9.

The normalized anastomotic gap *x*_*net*_/2*L* depends on the product of four dimensionless parameters, namely *aE*_*s*_/*E*_*L*_, *L*/*l*_*s*_, *N*_*s*_*r*_*s*_/*R*, *r*_*s*_/*h*, abbreviated herein as *P*_4_, and suture pre-tension parameter . However, utilizing plausible range of parameter values, we observe that the contribution of *r*_*s*_/*h* is relatively small. Figure 5 plots the normalized gap as a function of the normalized radial displacement *u*/*R*, for different values of the product *P*_4_, assuming zero suture pre-tension.

Although Figure 5 presents the variation of the anastomotic gap in a mathematically complete way, we opted to provide this information in Figures 6 and 7 in a more elaborate and practically appealing manner, in terms of the design parameters *aE*_*s*_/*E*_*L*_, *L*/*l*_*s*_, *A*_*s*_/*hR*, *N*_*s*_, in order to provide simpler and useful graphs for the optimal selection of materials and improved functionality of sutures. In particular, Figures 6 and 7 highlight the influence of the variation of the suture stiffness (Figures 6(a,b)), the stitch length (Figures 6(c,d)), the suture cross-section area (Figures 7(a,b)), and the number of stitches (Figures 7(c,d)) on the anastomotic gap, for two different sets of parameters. The results suggest that increasing the value of any of the design parameters yields a decreased anastomotic gap. In particular, the most influential parameter in drastically reducing the anastomotic gap is the number of utilized stitches, *N*_*s*_, as can be seen from Figures 7(c,d).

Figure 8 plots the normalized tensile force in each stitch (suture strain) as a function of the normalized radial displacement for different values of parameters 2*L*/*l*_*s*_, *aE*_*s*_/*E*_*L*_ and *N*_*s*_ (assuming ). It can be observed that the normalized suture tensile force is decreased as the number of stitches is increased, whereas the ratio of suture-to-artery elastic modulus and the normalized stitch length do not affect significantly the tensile force developed in each stitch. The latter is also true for the suture radius as suggested by equation (23).

Figure 9 plots the normalized embedding stress due to suture-artery contact interaction as a function of the normalized radial displacement for different values of parameters *aE*_*s*_/*E*_*L*_ and *N*_*s*_. It can be seen from Figure 9 that in order to reduce the embedding stress, the number of stitches must be increased, whereas the parameter *aE*_*s*_/*E*_*L*_ plays an insignificant role. Moreover, the embedding stress becomes smaller with increasing suture radius, as can be seen from equation (24).

It should be noted that, for a typical anastomosis scheme (with parameters within the physiological range) and for , when the value of pre-tension  exceeds a certain value (derived from ) the arterial wall is likely to fail. On the other hand, for lower values of pre-tension and for , the application of suture pre-tension can result in reducing the anastomotic gap (equation (22)), while not affecting considerably the embedding stress (equation (23)) and suture tensile force (equation (24)).

### Design considerations

For design purposes the peak values of response are considered. For the general case where , the failure scenarios discussed previously can be prevented by recasting the inequalities (10), (11), (12) in the form:
252627

where *u*_max_ stands for the maximum radial displacement of artery, *d*_*rbc*_ is the red blood cell diameter (approximately equal to 7 μm), and *f*_*s*,*u*_, *σ*_*s*,*u*_ are known from the suture strength and the tensile strength of the arterial wall, respectively. The right-hand side of inequalities (25) to (27) denotes the minimum number of stitches required to prevent suture failure, arterial-wall tearing, and development of excessive gap, respectively. Obviously, the final selection will be the maximum of *N*_1_, *N*_2_, *N*_3_. However, the number of stitches should not violate the geometric constraint of equation (13), which practically can be stated as
28

Therefore, the final selection of number of stitches should be bounded by
29

Failure to satisfy inequality (29) means that the material selection and geometric parameter must be rethought. Typical values related to suture materials indicate that *N*_1_ < *N*_2_ or *N*_3_, although deteriorated stitches as well as the presence of sutures knots can change this.

When the suture strength is larger than the knot strength, the stitches will fail on the knot region, otherwise the failure will occur elsewhere. Experiments on the mechanical properties of different suture materials were performed by Brouwers et al. [25]. Table 1 reports values for the tensile strength of plain sutures and the tensile strength range for seven knots under dynamic loading. Moreover, the arterial longitudinal strength was found to be between 1–3 MPa, based on dynamic biaxial tension tests on human aortic tissues [19].

**Table 1:** **Tensile strength of untied and tied fiber (Based on Brouwers et al.** [25]**)**

Suture material	Diameter (mm)	Suture strength (N)	Knot strength (N)
Plain catgut	0.36	25.5	23.7 - 29.6
Maxon	0.31	34.5	22.1 - 46.1
PDS	0.3	27.2	12.4 - 36.5
Prolene	0.26	16.7	6.2 - 26.7
Dexon	0.24	29.1	24.1 - 39.4
Mersilene	0.26	28.3	20.5 - 37.8
Vicryl	0.29	34.6	14.1 - 38.8

Note that, for the particular case where , the derived inequalities (25) to (27) are not valid. In this case, the potential failure is not dependent on the number of sutures *N*_*s*_, but rather on whether the pre-tension exceeds either the suture strength or artery strength.

### Numerical example

To illustrate the applicability of the proposed analytical model, a design example is presented, in which the minimum number of stitches required to prevent suture failure, arterial-wall tearing, and development of excessive anastomotic gap, is calculated for a given set of typical artery and suture parameters (Table 2). More values for the mechanical properties of human ascending thoracic aorta can be found in the study of Gozna et al. [28].

**Table 2:** **Parameters used in numerical example**

Parameter	Value
*Artery*	
Length, *L* (cm)	3
Radius, *R* (cm)	0.6
Thickness, *h* (cm)	0.11
Arterial tissue density, *ρ* (kg m^−3^)	1160
Initial displacement, *u*_0_ = (2/3)*u*_*st*_ (mm)	0.499
Circumferential Young’s modulus, *E*_*θ*_ (kPa)	700
Longitudinal Young’s modulus, *E*_*L*_ (kPa)	400
Tissue strength, *σ*_*s*,*u*_ (MPa)	3
Red blood cell diameter, *d*_*rbc*_ (μm)	7
*Suturing (Continuous, Prolene)*	
Length, *l*_*s*_ (cm)	0.2
Radius, *r*_*s*_ (mm)	0.13
Young’s modulus, *E*_*s*_ (GPa)	1.5
Participation factor, *a*	1.7
Suture pre-tension, (N)	0
Suture strength, *f*_*s*,*u*_ (N)	16.7
*Loading*	
Systolic pressure, *p*_*s*_ (mmHg)	120
Diastolic pressure, *p*_*d*_ (mmHg)	80
Systolic duration, *t*_*s*_ (sec)	0.35
Cardiac pulse duration, *t*_*cp*_ (sec)	1

Based on these parameter values, the maximum arterial response, occurring during the systolic phase, is calculated as *u*_max_ = 0.997 mm. The maximum circumferential strain *ϵ*_*θ*,max_ = *u*_max_/*R* = 16.6% is within the validity range of the small-deformation assumption. Based on inequality (29), the optimal selection of the number of stitches for this example is *N*_*s*_ = 17. For the selected value of the design parameter *N*_*s*_, the response quantities of interest are derived as: suture force *f*_*s*_ = 0.24 N (< 16.7 N ≡ *f*_*s*,*u*_), embedding stress *σ*_*s*_ = 1.43 MPa (< 1.5 MPa ≡ *σ*_*s*,*u*_/2), and anastomotic gap *x*_*net*_ = 6.02 μm (< 21 μm ≡ 3*d*_*rbc*_). As expected, by virtue of satisfying simultaneously the objective functionalities given by equations (10), (11), (12), all response quantities fall within the accepted range of values, preventing any of the aforementioned failure scenarios. Nevertheless, the calculated embedding stress is marginally acceptable, and the slightest increase of its value may lead to arterial-wall tearing. That is, despite the fact that the suture can withstand tensile force up to 16.7 N, any suture pre-tension  applied by the surgeon in tying the knot may cause arterial injury.

### Validation of the model

The present model is fully analytic and has been conceived to be simple with minimum computational costs, and hence suitable for potential clinical application. The model incorporates a plethora of the most important-to-the-surgeon parameters for the first time, at the expense however of strong simplifying hypotheses. One main simplification is the linearization of the mechanical response of the anastomosis walls. Moreover, anisotropy in the circumferential and longitudinal direction has been retained also in an approximate way, ignoring Poisson effects. In addition, failure criteria based on octahedral equivalent stresses may not be completely appropriate for describing the strength of the arterial tissue. Finally, a limit-state analysis has been adopted for the failure mechanism of arterial tissues subject to the loading condition imposed by the stitches.

The aforementioned issues, important by themselves, do not change the holistic view of the present paper. The linearization of all presented responses gives consistent strains of the order of 20%. The use of more elaborate hyperelastic constitutive laws does not change appreciably the central results of our work. Linear-elasticity estimates can be adjusted by appropriate changes of the elastic moduli. Poisson effects can reduce the stitching results by about 30%, thus ignoring the Poisson effects is not against safety. Failure of the arterial walls is still an uncharted topic. It is most probable that failure depends on energy criteria, and in this respect the shear stress used in this work corresponds to a critical deviatoric energy. Finally, the limit-state analysis based on a critical shear stress can be easily recast into a tearing criterion based on the almost-uniaxial state of stress on the sides of the stitches (the linear-elasticity local model predicts a stress concentration factor of about two).

Although the literature contains several experimental studies dealing with the compliance of the anastomotic region [9–13], we found that many parameters that seem to affect the suture stressing are not reported (e.g. the number of stitches *N*_*s*_). Our present work indicates that more details regarding the suture material and suturing technique should be reported, especially if the para-anastomotic hypercompliant zone (PHZ) phenomenon needs to be addressed. Previous experimental studies of end-to-end anastomosis between isocompliant arteries or grafts investigate the compliance of the anastomotic region, whereas the main response quantities calculated in this study (*x*_*net*_, *f*_*s*_, *σ*_*s*_) are not reported in experimental studies. Nevertheless, it is shown that the present study provides a good estimation of the compliance value of the anastomotic region with respect to the published experimental results.

Compliance (*C*) is the circumferential strain of the systolic phase in respect to the strain of the diastolic phase *ϵ*_*sd*_ divided by the pressure difference:
30

where *D*_*s*_ and *D*_*d*_ are the arterial diameters under systolic and diastolic pressure, respectively. Hasson et al. [10, 11] calculated the compliance of dog arterial grafts under dynamic loading. The compliance away from the PHZ was 0.06% mmHg^−1^ for the first study and 0.05% mmHg^−1^ for the later study. Ulrich et al. [13] calculated the compliance of pig arterial grafts under dynamic loading as 0.075% mmHg^−1^. The calculated compliance of our numerical example is 0.12% mmHg^−1^. Given that the mechanical data and pressure profile data were not available for most of the experimental studies and that our model is subjected to pulse loading of the first loading cycle (meaning that the calculated displacements may be up to two times larger than the static or long-term dynamic loading), our model constitutes a good approximation of the experimental results.

**Figure 10:**
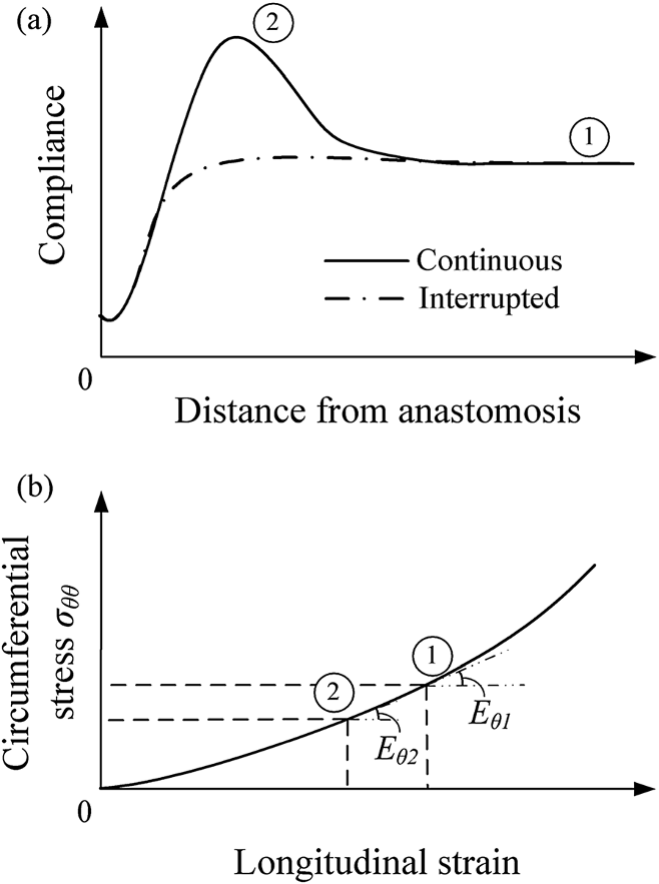
**Schematic correlation of PHZ phenomenon to the stiffness of the arterial tissue. (a)** Compliance of the anastomotic region. The PHZ phenomenon (region 2) exists in the case of continuous stitches, as was concluded by Hasson et al. [11]. Away from the anastomosis zone the compliance is constant (region 1), **(b)** Circumferential stress-longitudinal strain relationship of a nonlinear hyperelastic material. In the case of continuous stitching, the longitudinal stretch is lower than that of the discrete stitching. This results to a lower tangential modulus (*E*_*θ*1_ > *E*_*θ*2_), implying a higher compliance at the PHZ.

Of particular interest is the PHZ phenomenon. Hasson et al. [11] found that the PHZ phenomenon occurs more frequently for anastomosis of the continuous stitching technique than the interrupted stitching technique. Figure 10(a) shows the schematic compliance along the anastomotic region. The PHZ phenomenon (region 2) is pronounced in the case of continuous stitches, whereas away from the anastomosis zone the compliance is constant (region 1). From our study, the net gap *x*_*net*_ is increased by 15% in the case of continuous stitching compared to the case of interrupted stitching. This may justify the decreased longitudinal stretch Δ*l*/*L* and lower tangent elastic modulus *E*_*θ*1_ > *E*_*θ*2_ (Figure 10(b)) of the continuous stitching case. The decrease of tangent elastic modulus results to higher compliance at the PHZ.

The experimental results suggest a decrease of stiffness by about 29% [11]. From the numerical example presented in our study, the total longitudinal stretch away from the suture line is 1.36. The longitudinal stretch at the PHZ is reduced by 29% compared to the longitudinal stretch away from the anastomotic region. Taking a nonlinear constitutive law according to Skalak et al. [29], the continuous stitching (stretch 1.27) decreases the tangent modulus by 24% in comparison to the interrupted stitching (stretch 1.36), indicating that the increase of compliance at the PHZ can be correlated to the decrease of stiffness, as Hasson et al. [11] suggest.

In conclusion, the present model, even though simple and approximate, captures adequately the essence of the phenomenon. More complex models can be important in refining the present results, but on the other hand will require more material data that may be difficult to obtain or assess their direct contribution.

## Conclusions

Presented in this study is the mathematical formulation governing the dynamic behavior of end-to-end arterial anastomosis, with emphasis on suture-artery interaction and the axial-circumferential deformation coupling in the artery response. Closed-form, time-dependent expressions were derived for the system response, in terms of the radial displacement of artery (equation (4)), the anastomotic gap (equation (20)), the suture tensile force (equation (17)), and the embedding stress due to suture-artery contact interaction (equation (21)). It is worth noting that, although linear elastic, the model is comprehensive in that it captures the effects of all pertinent parameters, including the geometric and mechanical properties of sutures and arterial walls, the number of sutures, the loading characteristics, the longitudinal residual stresses, and suture pre-tensioning. As a result, the response was obtained as a function of as many as seventeen input parameters (*L*, *R*, *N*_*s*_, *h*, *E*_*θ*_, *E*_*L*_, *p*_*s*_, *p*_*d*_, *t*_*s*_, *t*_*cp*_, *ρ*, *u*_0_, *l*_*s*_, *E*_*s*_, *r*_*s*_, *a*, ). Nevertheless, on normalizing appropriately the response quantities, the problem can be described by only five dimensionless parameters (*aE*_*s*_/*E*_*L*_, *L*/*l*_*s*_, *N*_*s*_*r*_*s*_/*R*, *r*_*s*_/*h*, ).

Inherent in the analysis are limitations stemming from the underlying model assumptions (as discussed in section *Methods*). We are currently working on similar problems where we gradually relax assumptions made regarding the artery cylindrical geometry, the material linear constitutive relations, the arterial-wall homogeneity, and the kinematic conditions.

Findings obtained by the suture-tissue interaction analysis reveal the nonlinear dependency of the system response on the radial extension of artery and highlight useful interrelations among the problem parameters. In regard to the normalized anastomotic gap, the results suggest that increasing the value of any of the design parameters, excluding , yields a decreased anastomotic gap. In particular, the most influential parameter in drastically reducing the anastomotic gap is the number of utilized stitches, *N*_*s*_, as can be seen from Figures 7(c,d). The normalized suture tensile force is instead affected only by the number of stitches. A higher number of utilized stitches results in a smaller tensile force developed in each stitch (Figure 8). It has also been shown that the normalized embedding stress is decreased as the number of stitches is increased, whereas the influence of the ratio of suture-to-artery elastic modulus on the embedding stress is insignificant (Figure 9).

It should be noted that, for a typical anastomosis scheme (with parameters within the physiological range) and for , when the value of pre-tension  exceeds a certain value (derived from ) the arterial wall is likely to fail. On the other hand, for lower values of pre-tension and for , the application of suture pre-tension can result in reducing the anastomotic gap (equation (22)), while not affecting considerably the embedding stress (equation (23)) and suture tensile force (equation (24)).

In conclusion, the primary contribution of this study is the development of a fundamental analytical model that predicts the dynamic behavior of end-to-end arterial anastomosis. Derived from first principles, thus characterized by generality, the proposed model offers new and better-substantiated understanding of the mechanics of end-to-end anastomosis scheme. The mathematical formulation reveals useful interrelations among the problem parameters, thus making the proposed model a valuable tool for the optimal selection of materials and improved functionality of sutures. The comprehensive failure criteria established in this study can ultimately form the basis for the development of vascular anastomosis guidelines pertaining to the prevention of post-surgery implications.

## Appendix A

### A1: proof of equation (1)

By considering the equilibrium of the element of the circular ring with unit length shown in Figure 1 we obtain
a.1

where *m* = *ρRhdθ* is the mass of the arterial element of unit length. From Hooke's law, the axial force *N* is given by
a.2

On substituting the above expression in equation (a.1), and by considering small angles (so that sin(*dθ*/2) ≈ *dθ*/2), we obtain
a.3

By dividing by *Rdθ* we obtain the equation governing the radial displacement response as
a.4

### A2: proof of equation (4)

The total response of the system to a pulse loading with nonzero initial conditions is the sum of the response to the pulse loading *u*_*p*_(*t*) and the response to free vibration *u*_*f*_(*t*) due to initial conditions.

The response to free vibration with initial displacement *u*_*f*_(0) = *u*_0_ and initial velocity  is given by
a.5

The response of the system to impulse force *p*(*t*) can be determined by using the convolution (Duhamel) integral. A convolution integral is simply the integral of the product of the external force *p*(*τ*) and the unit-impulse response function of the system *h*(*t* − *τ*):
a.6

The response in the systolic phase (0 ≤ *t* ≤ *t*_*s*_), in which the system is subjected to constant force *p*(*τ*) = *p*_*s*_, is calculated as
a.7

The response in the diastolic phase (*t*_*s*_ ≤ *t* ≤ *t*_*cp*_), in which the system is subjected to impulse force *p*(*τ*) = *p*_*s*_ − (*p*_*s*_ − *p*_*d*_)(*τ* − *t*_*s*_)/( *t*_*cp*_ − *t*_*s*_), is derived from
a.8

in which the first term concerns the force-vibration response associated with the diastolic phase loading, and the last two terms concern the free-vibration response due to initial conditions *u*_*p*_^*I*^(*t*_*s*_) and  induced at the end of the systolic phase. On carrying out the calculations, equation (a.8) simplifies to
a.9

The total displacement is then obtained as the sum of the pulse-loading response *u*_*p*_(*t*) and the free-vibration response *u*_*f*_(*t*):
a.10

### A3: proof of equations (**6**), (**7**), (**8**), (**9**)

The maximum displacement of the arterial system may occur either during the systolic phase (0 ≤ *t* ≤ *t*_*s*_) or during the diastolic phase (*t*_*s*_ < *t* ≤ *t*_*cp*_). The maximum displacement of the systolic phase (for |*u*_0_|/*u*_*st*_ < 1) occurs for cos *ω*_*n*_*t* = − 1. Therefore, by substituting this expression into the first part of equation (4) the maximum displacement of the systolic phase  is obtained as
a.11

To calculate the time instant *t*_1_ corresponding to the maximum response of the diastolic phase, the derivative of the displacement with respect to time is set equal to zero:
a.12

which can be recast in the following form
a.13

where
a.14a.15a.16

On solving for *t*_1_ we get the time instant corresponding to the maximum response of the diastolic phase as
a.17

The maximum displacement of the diastolic phase  is calculated at *t* = *t*_1_ as
a.18

The overall maximum response is then obtained as
a.19

### A4: proof of equation (**19**)

The equilibrium of forces in the axial direction requires that
a.20

Substituting equations (17) and (18) into equation (a.20), the equilibrium of forces in the axial direction yields
a.21

By combining equations (15) and (16), the net gap between the anastomosed artery segments is derived as
a.22

Substituting equation (a.22) into equation (a.21), the equilibrium equation is expressed in terms of Δ*l*(*t*) as
a.23

which can be readily solved for the tensile deformation:
a.24

## Appendix B: Solution of end-to-end anastomosis between artery and graft material

**Figure 11:**
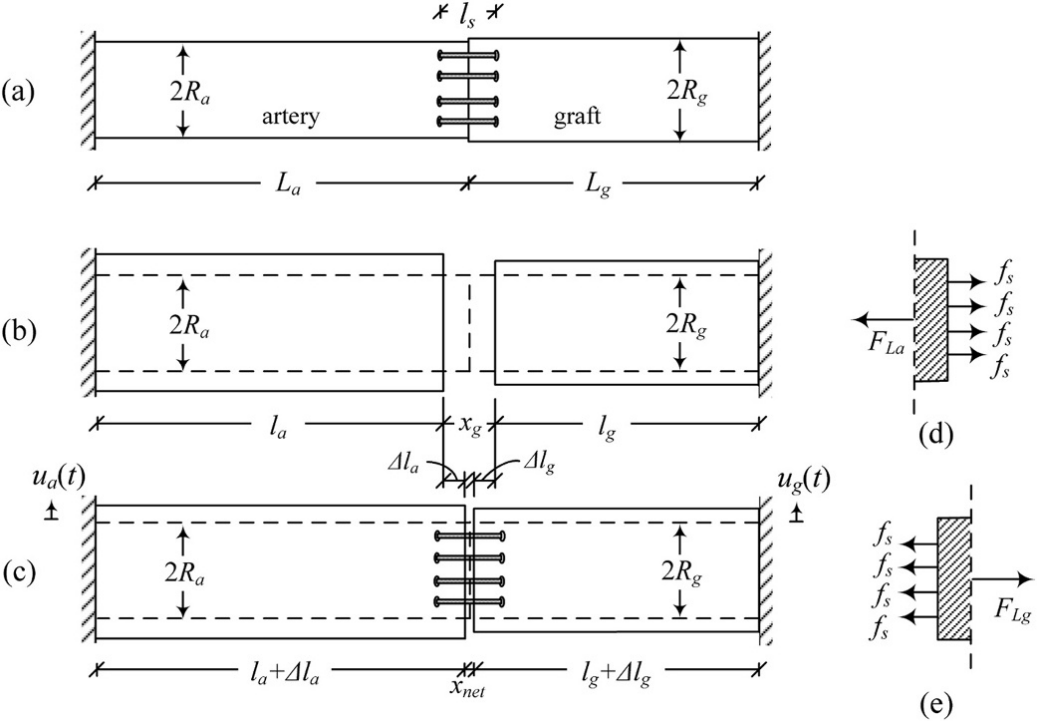
**Artery-graft end-to-end anastomosis analysis. (a)** Anastomosis model (at-rest state); the artery and graft are clamped at the far ends and no pressure is transmitted at this stage since the artery is emptied from the blood, **(b)** unrestrained deformed state (without sutures); the blood volume is conserved, **(c)** deformed state of anastomotic region due to dynamic loading, **(d)** forces acting on end-element of artery segment, **(e)** forces acting on end-element of graft segment.

This section presents the general solution of an end-to-end anastomosis between a host artery and a graft, each one having different geometrical and mechanical properties. The artery segment has length *L*_*a*_, radius *R*_*a*_, thickness *h*_*a*_, and Young’s modulus in the longitudinal direction and circumferential direction *E*_*La*_ and *E*_*θa*_, respectively, whereas the graft has length *L*_*g*_, radius *R*_*g*_, thickness *h*_*g*_, and Young’s modulus in the longitudinal direction and circumferential direction *E*_*Lg*_ and *E*_*θg*_, respectively (Figure 11(a)). The conservation of the blood volume requires that the artery initial length *L*_*a*_ decrease to *l*_*a*_ and the graft initial length *L*_*g*_ decrease to *l*_*g*_ (Figure 11(b)) according to:

b.1b.2

where *u*_*a*_ and *u*_*g*_ are the radial deformations of the artery and graft, respectively. Note that the graft has not initial radial displacement due to residual stresses. The gap developed in the unrestrained (without sutures) state of the artery is determined as
b.3

Therefore, the resulting net gap developed in the restrained (with sutures) anastomotic region can be derived from
b.4

where Δ*l*_*a*_ is the tensile deformation due to the artery/stitches interaction, and Δ*l*_*g*_ is the tensile deformation due to the graft/stitches interaction (Figure 11(c)).

The tensile forces developed in the suture, arterial tissue, and graft are given respectively by
b.5b.6b.7

The unknown tensile deformations Δ*l*_*a*_ and Δ*l*_*g*_ can be derived from equilibrium of forces in the axial direction, *F*_*La*_(*t*) = *F*_*Lg*_(*t*) and *F*_*La*_(*t*) = *aN*_*s*_*f*_*s*_(*t*) (Figure 11(d,e)), yielding
b.8b.9

Substituting equations (b.3), (b.8) and (b.9) into equation (b.4), we obtain the net gap between the anastomosed artery segments as
b.10

Note that a gap across the anastomotic interface will be formed only if the tension developed in the arterial tissue exceeds the total suture pre-tension. The suture tensile force *f*_*s*_ developed in each stitch can be obtained from equation (17). The embedding stresses induced on the arterial wall *σ*_*sa*_ and graft wall *σ*_*sg*_ must be compared to the strength of the artery *σ*_*sa*,*u*_ and strength of the graft *σ*_*sg*,*u*_ respectively:
b.11b.12
